# Mitigation of Methimazole-Induced Hepatic Injury by Taurine in Mice

**DOI:** 10.3797/scipharm.1408-04

**Published:** 2014-09-30

**Authors:** Reza Heidari, Akram Jamshidzadeh, Nahid Keshavarz, Negar Azarpira

**Affiliations:** 1 Pharmaceutical Sciences Research Center, Shiraz University of Medical Sciences, 7146864685 Shiraz, Iran; 2 Pharmacology and Toxicology Department, Shiraz University of Medical Sciences, 7146864685 Shiraz, Iran; 3 Transplant Research Center, Shiraz University of Medical Sciences, 7146864685 Shiraz, Iran

**Keywords:** Antithyroid, Endocrinology, Hepatoprotective, Drug-induced liver injury (DILI)

## Abstract

Methimazole is the most widely prescribed antithyroid medication in humans. However, hepatotoxicity is a deleterious adverse effect associated with methimazole administration. No specific protective agent has been developed against this complication yet. This study was designed to investigate the role of taurine as a hepatoprotective agent against methimazole-induced liver injury in mice. Different reactive metabolites were proposed to be responsible for methimazole hepatotoxicity. Hence, methimazole-induced liver injury was investigated in intact and/or enzyme-induced animals in the current investigation. Animals were treated with methimazole (200 mg/kg, by gavage), and hepatic injury induced by this drug was investigated in intact and/or enzyme-induced groups. Markers such as lipid peroxidation, hepatic glutathione content, alanine aminotransferase (ALT) and alkaline phosphatase (ALP) in plasma, and histopathological changes in the liver of animals were monitored after drug administration. Methimazole caused liver injury as revealed by increased plasma ALT. Furthermore, a significant amount of lipid peroxidation was detected in the drug-treated animals, and hepatic glutathione reservoirs were depleted. Methimazole-induced hepatotoxicity was more severe in enzyme-induced mice. The above-mentioned alterations in hepatotoxicity markers were endorsed by significant histopathological changes in the liver. Taurine administration (1 g/kg, i.p.) effectively alleviated methimazole-induced liver injury in both intact and/or enzyme-induced animals.

## Introduction

Drug-induced liver injury (DILI) is a major problem in the pharmaceutical industry and many drugs are known to have adverse effects toward the liver [1, 2]. Thioamides are the leading medications used in the treatment of hyperthyroidism in humans [3]. Methimazole is the most convenient thioamide drug against hyperthyroidism [4]. Several adverse events are attributed to methimazole administration, including deleterious ones such as agranulocytosis and hepatotoxicity [5–7]. Although several cases of methimazole-induced hepatic injury were reported [6–10], no specific protective agents have been developed against this complication so far. Different mechanisms have been proposed to be involved in methimazole-induced liver injury. Reactive metabolites formation [11–13], oxidative stress induction [13–15], and intracellular organelle dysfunction [13, 16] seem to play a role in methimazole-induced hepatotoxicity.

Taurine (2-aminosulfonic acid) is a non-essential amino acid found in the daily dietary intake of humans [17]. A wide range of different physiological roles are attributed to taurine [18] including neuroprotective [19], antiepileptic [20], cardio-protective [21], and protection against diabetes-related complications [22, 23]. Furthermore, it has been found that this amino acid protected the liver from xenobiotic-induced hepatotoxicity, including different drugs [24–30]. The mechanisms by which taurine provide protection are many and varied. It has been found that this amino acid has antioxidant and radical scavenging properties [31–33]. Taurine plays a role as a cellular osmoregulator [34] and prevents increases in calcium ions (Ca^2+^) [35]. Calcium serves as a critical intracellular ion involved in cell death processes [35]. The antioxidant properties and the effects of this amino acid on different cellular defense mechanisms, such as glutathione peroxidase (GPx), glutathione transferase (GST), catalase (CAT), and superoxide dismutase (SOD), were proven in previous investigations [36, 37]. Some studies have shown that taurine effectively protected crucial intracellular organelles such as the mitochondria against stress [35, 38, 39]. Several other investigations revealed that taurine was able to modulate immune system-mediated toxicities [40]. All these unique properties of taurine, in addition to its safety even in very high doses [41–43], make this amino acid a potential therapeutic option against xenobiotic-induced toxicity in different organs, including drug-induced liver injury (DILI). Moreover, previously we have shown that taurine effectively prevented methimazole-induced cytotoxicity in an *in vitro* model of isolated hepatocytes [39].

This investigation attempted to evaluate the role of this amino acid against methimazole-induced hepatic injury in mice. The hepatoprotective effects of the taurine study were compared with N-acetylcysteine (NAC) as a gold standard treatment of drug-induced hepatic injury [44]. Furthermore, it has been shown that NAC effectively mitigated methimazole-induced liver injury in mice [45].

## Results and Discussion

**Fig. 1:**
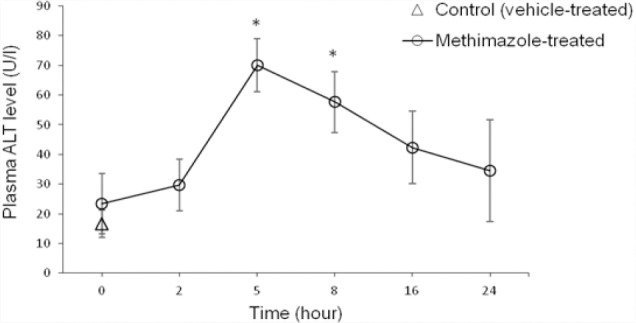
Methimazole-induced changes in plasmatic ALT levels over time. Data are expressed as Mean±SEM for six mice. Methimazole (200 mg/kg) was administered by gavage and plasma ALT level was assessed at different time points. * Indicates significantly higher than the control group (P<0.05)

Methimazole was administered at different doses (50, 100, and 200 mg/kg, oral) and plasma ALT was monitored at different time intervals (Figure 1). It was found that 200 mg/kg of methimazole caused a peak in plasmatic ALT levels (>3 ULN, which indicates hepatic injury [46]), 5 hours after drug administration (Figure 1). This elevation in ALT was significantly higher than the control (vehicle-treated) animals (Figure 1). The appropriate toxic dose and time for future experiments was obtained through this estimation (200 mg/kg after 5 hours) (Figure 1). Previously, we showed that methimazole administration to mice (100 mg/kg, i.p.) also increased plasmatic ALT levels, but was accompanied by slight histopathological changes in the animals’ livers [45].

**Fig. 2:**
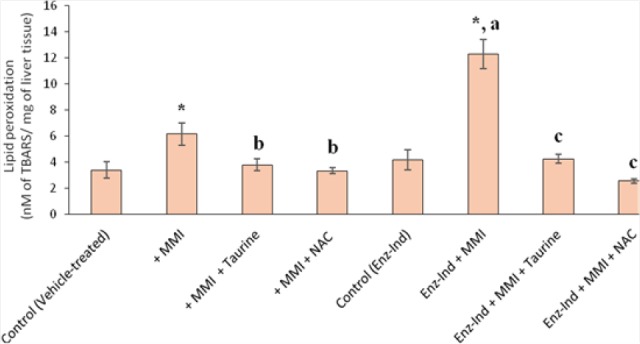
Methimazole-induced lipid peroxidation in mice livers. Data are expressed as Mean±SEM for six animals in each group. Mice were treated with methimazole (200 mg/kg, orally) and the level of lipid peroxidation was assessed after 5 hours of drug administration. MMI: methimazole, Enz-Ind: enzyme-induced animals. * Significantly higher than the control group (P<0.05). ^a^ Significantly higher than the MMI-treated group (P<0.05). ^b^ Significantly lower than the MMI-treated group (P<0.05). ^c^ Significantly lower than the enzyme-induced group which was treated with MMI (P<0.05)

**Fig. 3:**
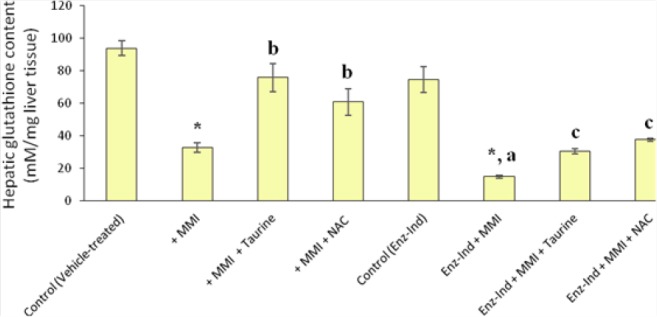
Effect of methimazole on hepatic glutathione reservoirs in mice. Data are given as Mean±SEM for six mice. MMI: methimazole, Enz-Ind: enzyme-induced animals. * Significantly higher than the control group (P<0.05). ^a^ Significantly higher than the MMI-treated group (P<0.05). ^b^ Significantly lower than the MMI-treated group (P<0.05). ^c^ Significantly lower than the enzyme-induced group which was treated with MMI (P<0.05)

**Fig. 4:**
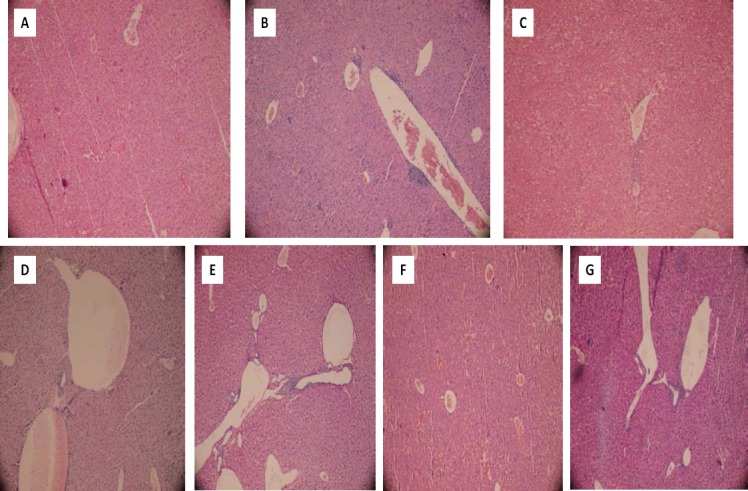
Histopathological changes in mice livers after methimazole administration in normal and/or enzyme-induced animals. Haematoxylin and eosin (H&E) staining. A: control (vehicle-treated), B: +methimazole (200 mg/kg), C: enzyme-induced animals + methimazole (200 mg/kg), D: methimazole + taurine (1 g/kg), E: methimazole + NAC (300 mg/kg), F: enzyme-induced + methimazole (200 mg/kg) + taurine (1 g/kg), G: enzyme-induced + methimazole (200 mg/kg) + NAC (300 mg/kg). Methimazole administration caused sinusoidal congestion and slight inflammatory cell infiltration in mice livers (B). When enzyme-induced animals were treated with methimazole, liver necrosis, inflammation, and hemorrhage occurred (C). Administration of taurine (D & F) and/or NAC (E & G), effectively alleviated methimazole-induced histopathological changes in the liver in both intact and/or enzyme-induced animals.

It was found that methimazole (200 mg/kg, by gavage) caused hepatic damage in mice as revealed by increases in plasmatic ALT levels (Table 1). Moreover, a significant increase in the thiobarbituric acid reactive substances (TBARS) level of liver tissue (Figure 2) and a decrease in liver glutathione content (Figure 3) were detected in the drug-treated animals. A sharp increase in ALT activity was observed in the enzyme-induced mice after methimazole administration (Table 1). When enzyme-induced animals were treated with methimazole, a high level of lipid hydroperoxides (Figure 2) and an intense decline in hepatic glutathione levels (Figure 3) were detected. Two out of six enzyme-induced animals were dead when they were treated with methimazole (Table 1). This might indicate the crucial role of reactive metabolite(s) in mediating methimazole-induced hepatotoxicity. A slight increase in inflammatory cell infiltration in the pre-portal and pre-central regions after a histopathological evaluation of methimazole-treated mice liver was evidenced (Figure 4, part B). Extensive necrosis of the liver occurred when the enzyme-induced mice were treated with methimazole (Figure 4, part D).

**Tab. 1:**
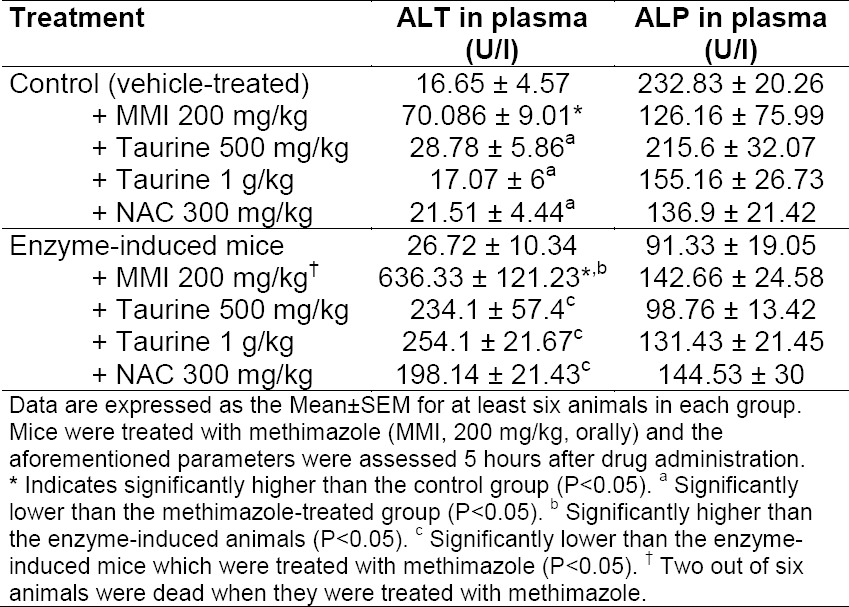
Effects of taurine and NAC on plasmatic biochemical parameters related to methimazole-induced liver injury in mice

**Fig. 5:**
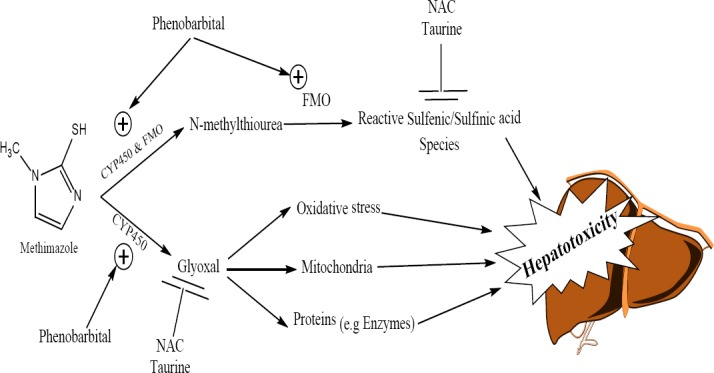
The proposed hepatoprotective mechanisms of taurine and/or N-acetylcysteine (NAC) against methimazole-induced liver injury. CYP450: cytochrome P450, FMO: flavin-dependent monoxygenases

The formation of potentially reactive metabolites plays a pathogenic role in the mechanism of DILI in most cases [52]. Glyoxal and N-methylthiourea are two suspected metabolites for methimazole-induced hepatic injury [12, 13] (Figure 5). Glyoxal is a well-known cytotoxic compound which induces oxidative stress, protein carbonylation, lipid peroxidation, and cellular glutathione reservoir depletion in hepatocytes [53, 54] (Figure 5). Previously, in an in vitro model of isolated rat hepatocytes, we found that glyoxal, as a metabolite which induced oxidative stress and affected mitochondrial function, might play a more predominant role in methimazole-mediated liver injury [13].

Different doses of taurine (250, 500, and 1000 mg/kg, i.p) were administered to find an appropriate hepatoprotective dose of this amino acid against methimazole-induced hepatic injury. It was found that the administration of 1g/kg of taurine effectively alleviated all the toxicity markers of methimazole-induced liver injury in mice. The hepatoprotective properties of taurine (1 g/kg, i.p.) against methimazole-induced hepatic injury was re-vealed by suppressing lipid peroxidation (Figure 2), preventing liver glutathione depletion (Figure 3), and a significant decrease in plasmatic ALT levels (Table 1). Moreover, this amino acid (1 g/kg) mitigated histopathological changes in the liver caused by methimazole in both intact and/or enzyme-induced animals (Figure 4). NAC administration (300 mg/kg, i.p.), effectively alleviated all hepatic adverse events associated with methimazole in this investigation (Table 1) (Figures 1–4).

The plasmatic ALT level is a gold standard marker for evaluating drug-induced hepatotoxicity [47]. On the other hand, the elevation in plasmatic ALP levels indicates the cholestatic nature of hepatic injury induced by xenobiotics [47, 48]. As previously reported, methimazole-induced liver injury is mostly the cholestatic type [49, 50], however, our data obtained from plasmatic ALP levels after methimazole administration (Table 1) didn’t show significant changes in ALP after methimazole administration.

The ALT elevation (>3 ULN) indicated xenobiotic-induced liver injury [51]. As we found in this study, plasmatic ALT levels were significantly elevated after methimazole administration, which indicated drug-induced liver injury (DILI). Taurine and/or NAC administration effectively diminished the elevation in ALT levels caused by methimazole due to their hepatoprotective properties (Table 1).

Our study on methimazole in enzyme-induced animals is in line with previous research which indicates the role of reactive intermediates in methimazole-induced liver injury [13, 26, 45]. Increases in lipid peroxidation and plasmatic ALT activities were more severe in enzyme-induced animals. These are indicators that endorse the role of reactive drug intermediates in methimazole-induced hepatotoxicity. Moreover, the massive necrosis of mice livers after methimazole administration (Figure 4) proves the role of reactive metabolites. Additionally, it has been shown that liver enzyme inhibition alleviated methimazole-induced hepatocyte injury [11, 13].

Although some previous investigations indicated that no significant histopathological changes accompanied methimazole administration [11], we have found that inflammatory cell infiltration occurred after methimazole administration to mice (Figure 4, part B). Some human case reports of methimazole-induced hepatic injury also reported the presence of inflammatory cells in liver biopsies taken from patients [6, 55]. These discrepancies might be due to the route and dose of the administered drug. As methimazole-induced hepatic injury is categorized as an idiosyncratic reaction of unpredictable nature, the hepatotoxic reactions might be expected after drug administration *in vivo*.

As mentioned, it has been shown that reactive metabolites, induction of oxidative stress, and intracellular organelle dysfunction [11–13, 16] seem to play a critical role in methimazole-induced injury toward hepatocytes. As taurine prevented hepatic glutathione depletion (Figure 3) and lipid peroxidation in mice livers (Figure 2), a part of the hepatoprotective effects of taurine against methimazole might be attributed to its role in attenuating oxidative stress induced by glyoxal as a methimazole reactive metabolite. Furthermore, taurine boosts cellular defense mechanisms and makes hepatocytes more resistant against toxic insults [56, 57]. Additionally, taurine plays as a mitochondrial-protecting agent [35]. Since this organelle might be an intracellular target where methimazole and its reactive intermediates could induce cellular dysfunction and toxicity [13, 39], the protecting properties of taurine might also be involved in the current investigation. NAC is a well-known glyoxal trapping agent [59, 60], and its hepatoprotective effect against methimazole might be attributed to its activity in scavenging reactive methimazole metabolites (Figure 5). The role of NAC in counteracting oxidative stress [61] and its effects on cellular glutathione status (Figure 3) can be other important factors.

The other methimazole metabolite, N-methylthiourea, is further metabolized to sulfenic and sulfinic acid species [58] (Figure 5). Sulfenic acids are reactive nucleophilic compounds, which are capable of interacting with different intracellular targets [59]. A part of the toxic effect of methimazole toward hepatocytes might be attributed to the reactive intermediates derived from N-methylthiourea [12, 60] (Figure 5). Hence, taurine might protect the liver from these reactive intermediates and prevent methimazole-induced hepatotoxicity (Figure 5).

As phenobarbital induces several types of cytochrome P450 (CYP450) and/or flavin-dependent monoxygenases (FMO) enzymes [61, 62], a higher level of reactive metabolites (glyoxal and N-methylthiourea) will be formed in enzyme-induced animals and consequently, methimazole-induced hepatotoxicity will be exacerbated (Figure 5). Further investigations on the specific enzyme responsible for converting methimazole to reactive intermediates may provide new strategies to prevent and/or treat methimazole-induced liver injury. Moreover, advanced cytotoxicity evaluation procedures like the assessment of hepatic inflammation or apoptosis might enhance our understanding of the mechanisms by which taurine protects cells against different xenobiotics.

Taken together, our data suggest that taurine possesses protective properties against methimazole-induced hepatic injury probably due to its effect on oxidative stress and its consequences such as lipid peroxidation and glutathione reservoir depletion. Moreover, the direct effect of taurine and NAC on methimazole reactive metabolites such as glyoxal and/or N-methylthiourea might also play a role in their hepatoprotective properties in mice.

## Experimental

### Chemicals

5,5’-Dithionitrobenzoic acid (DTNB) and *n*-butanol were purchased from Sigma-Aldrich (St. Louis, USA). Thiobarbituric acid (TBA) was obtained from SERVIA (Heidenberg, New York). Trichloroacetic acid (TCA), phenobarbital, and hydroxymethyl aminomethane (Tris), were purchased from Merck (Darmstadt, Germany). Methimazole was purchased from Medisca Pharmaceutique (Montreal, Canada). The kits for liver biochemistry analysis (ALT & ALP) were obtained from Pars Azmun Company (Tehran, Iran). All salts for preparing buffer solutions were of the highest grade commercially available.

### Animals

Male Swiss albino mice (25–35 g weight), were obtained from the Shiraz University of Medical Sciences (Shiraz, Iran). Mice were housed in cages on wood bedding at a temperature of 25±3°C. Animals had free access to food and water. Mice were randomly divided equally into 12 groups of six animals. Methimazole was administered orally (by gavage). Taurine and NAC were administered intraperitoneally (i.p.), 1 hour after methimazole administration. All agents were dissolved in 0.9% saline. The treatments were as follows:

1) Control; vehicle-treated (0.9% saline solution) only.
2) Methimazole (50 mg/kg).
3) Methimazole (100 mg/kg).
4) Methimazole (200 mg/kg).
5) Methimazole (200 mg/kg) + Taurine (250 mg/kg).
6) Methimazole (200 mg/kg) + Taurine (500 mg/kg,).
7) Methimazole (200 mg/kg) + Taurine (1g/kg).
8) Methimazole (200 mg/kg) + NAC (300 mg/kg).
9) Enzyme-induced mice (phenobarbital-pretreated mice, refer to “Experimental” section for more details).
10) Enzyme-induced mice + Methimazole (200 mg/kg).
11) Enzyme-induced mice + Methimazole (200 mg/kg) + Taurine (1g/kg).
12) Enzyme-induced mice + Methimazole (200 mg/kg) + NAC (300 mg/kg).

No significant toxicity with taurine and/or NAC was observed when administered alone in intact or enzyme-induced animals at the mentioned doses.

### Liver Glutathione Content

The glutathione contents of mice liver were assessed by determining the non-protein sulphydryl contents with the Ellman’s reagent [63]. Liver samples (200 mg) were homogenized in 8 mL of cooled EDTA solution (0.02 M). Five-mL of liver homogenate was mixed with 4 mL of distilled water and 1 mL of 50% trichloroacetic acid (TCA). The mixture was shaken and then centrifuged (765 g, 15 minutes, at 4°C). Then 2 mL of the supernatant was added to 4 mL of Tris buffer (pH= 8.9) and 100 µL of DTNB solution (0.01 M in methanol) [63]. The absorbance of the developed color was read at 412 nm using an Ultrospec 2000^®^ UV spectrophotometer.

### Lipid Peroxidation

The level of lipid peroxidation in mice livers was assessed by the thiobarbituric acid reactive substances test [64]. Briefly, the reaction mixture consisted of 0.5 mL of 10% liver homogenate, 3 mL phosphoric acid 1%, and 1 mL of 1% thiobarbituric acid (TBA). The mixture was shaken and then it was heated in boiling water (100°C) for 45 minutes. Four-mL of n-butanol was added to the reaction mixture after cooling and was vigorously shaken. After centrifugation in 765 g for 5 minutes, the absorbance of the developed color in n-butanol phase was read at 532 nm using an Ultrospec 2000^®^UV spectrophotometer.

### Plasma Biochemical Analysis and Liver Histopathology

Blood was collected from the abdominal *vena cava* under pentobarbital anesthesia, and the liver was removed. The blood was collected in EDTA-coated tubes and plasma was prepared by centrifugation. Alanine transaminase (ALT) and alkaline phosphatase (ALP) activities were measured in the plasma using commercial kits. For the histopathological evaluation, samples of the liver were fixed in formalin (10%). Paraffin-embedded sections of the liver were prepared and stained with haematoxylin and eosin before viewing with an Olympus CX21^®^ light microscope [65].

### Enzyme-Induced Mice

Animals were treated with phenobarbital (80 mg/kg, i.p. injection for three consecutive days) before the experiments [66]. At the fourth day, animals were treated with methimazole. No significant changes in plasma biochemistry and/or liver histopathology were detected when the animals were enzyme-induced by the aforementioned method.

### Data Analysis

Results are shown as the Mean±SEM for at least six animals. Comparisons between multiple groups were made by a one-way analysis of variance (ANOVA) followed by Tukey’s *post hoc* test. Differences were considered significant when p<0.05.
